# Trait‐based life strategies, ecological niches, and niche overlap in the nekton of the data‐poor Mediterranean Sea

**DOI:** 10.1002/ece3.6414

**Published:** 2020-05-26

**Authors:** Martha Koutsidi, Catherine Moukas, Evangelos Tzanatos

**Affiliations:** ^1^ Department of Biology University of Patras Patras Greece

**Keywords:** biological traits, ecological niche, fish, Mediterranean Sea, trait overlap

## Abstract

Biological traits can determine species ecological niches and define species responses to environmental variation. Species have a specific functional position in the biological community, resulting in interactions like interspecific competition. In this study, we used biological traits in order to define the life strategies of 205 nektonic species of the Mediterranean Sea. Furthermore, traits related to resource use were analyzed to determine the level of trait and niche overlap and their relationship to life strategies. Focusing on habitats of importance (Posidonia beds, coralligène formations, and lagoons), we investigated strategies and niches of the species present there. Finally, we examined the life strategy of Lessepsian species and investigated the niche overlap between them and indigenous species. Archetypal analysis indicated the existence of three life histories corresponding to strategies already documented for fish (equilibrium, periodic, and opportunistic), with some species also placed in intermediate positions. Niche overlap was evaluated by multiple correspondence analysis and the generation of a single distance metric between all species pairs. This identified species occupying relatively empty (underexploited) ecological niches, like the Lessepsian species *Siganus luridus* and *S. rivulatus*, a finding that can also be associated with their establishment in the Mediterranean. Most Lessepsian species were associated with the opportunistic life history strategy, again an important aspect related to their establishment. Also, we documented that most species occurring in important habitats have a relatively high overlap of niches. No significant differences were found in the life strategies across Mediterranean habitats; however, variation in niche overlap and traits related to habitat use was detected. The findings can be useful to determine theoretical competition between species and to identify empty ecological niches. Fisheries science can also benefit from comprehending the dynamics of competing stocks or predict the responses of data‐poor stocks to anthropogenic stressors from known examples of species with shared life strategies.

## Graphical abstract

In this study, we used biological traits in order to define the life strategies and niche overlap of 205 nektonic species of the Mediterranean Sea. Focusing on habitats of importance (Posidonia beds, coralligène formations, and lagoons), we investigated strategies and niches of the species present there. Finally, we examined the life strategy of Lessepsian species and investigated the niche overlap between them and indigenous species. We indicated the existence of three life histories (equilibrium, periodic, and opportunistic). Niche overlap was evaluated between all species pairs. This identified species occupying relatively empty ecological niches, like the species *Siganus luridus* and *S. rivulatus*. Most Lessepsian species were associated with the opportunistic life strategy, an aspect related to their establishment. Also, we documented that most species occurring in important habitats have a relatively high overlap of niches.

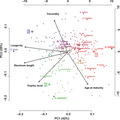

## 1 INTRODUCTION

Organisms possess various traits which relate to their ability to survive, grow, and produce offspring, that is, to increase their fitness (Violle et al., 2007). The term trait refers to the various aspects of the biology of an organism (e.g., physiology and behavior) that characterize its population responses to environmental changes or its role in ecosystem processes (Diaz & Cabido, 2001; Violle et al., 2007). Today, trait‐based approaches comprise an arsenal of methods using biological traits to describe ecological functioning (e.g., Bremner, Rogers, & Frid, 2006; Dolbeth, Vendel, Baeta, Pessanha, & Patrício, 2016; Naeem & Wright, 2003; Pecuchet et al., 2017; Villéger, Brosse, Mouchet, Mouillot, & Vanni, 2017).

The combinations of the various biological traits of a species form its life history strategy. Pianka (1970) has identified two main life strategies: The r‐strategy incorporates elements like fast growth, high fecundity, and mortality, while the K‐strategy conversely combines slow growth, low fecundity, and lower mortality rates. Regarding fish, Winemiller and Rose (1992) have indicated three strategies: the equilibrium, periodic, and opportunistic strategy. The life history strategies mentioned above allow fish and nektonic populations to exploit the resources available in their environment in the best possible way (Pianka, 2006); however, the limitation of resources in the ecosystem results in competition between different species. In fact, when the community is in balance, the combination of a species’ functional characteristics can describe its role in the community, thus its ecological niche (Cadotte, Arnillas, Livingstone, & Yasui, 2015; Violle & Jiang, 2009). In cases of niche overlap between different species, there is competition for the use of common resources (Hutchinson, 1957; Pianka, 2006).

As different species may sometimes serve the same functions (sometimes at similar or different rates—Duffy, Shackelton, & Holmes, 2008), an approach where species’ functional roles are examined using traits is of particular importance, especially in areas characterized by high biodiversity. The Mediterranean Sea is considered a biodiversity hotspot (Myers, Mittermeier, Mittermeier, da Fonseca, & Kent, 2000) as, despite its surface being lower than 0.7% of the global ocean, 4%–18% of global marine species are estimated to be distributed there (Bianchi & Morri, 2000). Furthermore, the Mediterranean is rapidly colonized by Lessepsian species (moving from the Red Sea to the Mediterranean across the Suez Canal—Por, 1978); thus, biodiversity patterns are rapidly changing. Arndt and Schembri (2015) have indicated that specific traits (like size, spawning type, and tendency to form schools) are related to the establishment and spread of Lessepsian fish species in the Mediterranean. The Mediterranean hosts a variety of habitats, some of which are characterized by a very high biodiversity, like *Posidonia oceanica* beds and coralligène formations. These habitats are widely distributed in the Mediterranean basin and have an important role in ecological processes providing shelter or being nursery areas (e.g., Ballesteros, 2006; Kalogirou, Corsini‐Foka, Sioulas, Wennhage, & Pihl, 2010). Lagoons are another important habitat type as, being eutrophic in the generally oligotrophic Mediterranean, they are highly productive, and act as fish nursery grounds (Viaroli et al., 1996). The examination of the biological traits of the species distributed in these habitats could provide information about their role and the interspecific relationships existing there which in turn may be useful for conservation efforts.

Despite the increasing number of publications about Mediterranean nekton (mainly fish), and the anthropogenic effects on nektonic populations, many aspects of the marine ecosystem are yet unknown and attempts to apply the so‐called Ecosystem Approach (Garcia, 2003) are relatively recent and exploratory (e.g., Link, Huse, Gaichas, & Marshak, 2020). In this data‐poor region, the definition of ecological niches and the determination of interspecific relationships are important challenges (Givan, Parravicini, Kulbicki, & Belmaker, 2017). While community assembly and interspecific relationships are generally investigated using abundance data of the species present in biological communities (e.g., Pecuchet, Törnroos, & Lindegren, 2016), an approach using bibliographic data on species traits could help indicate relationships that are otherwise neglected (because of the species' relative abundance) or highlight potential interactions that are hidden due to the particularities of the ecosystem studied.

**FIGURE 1:**
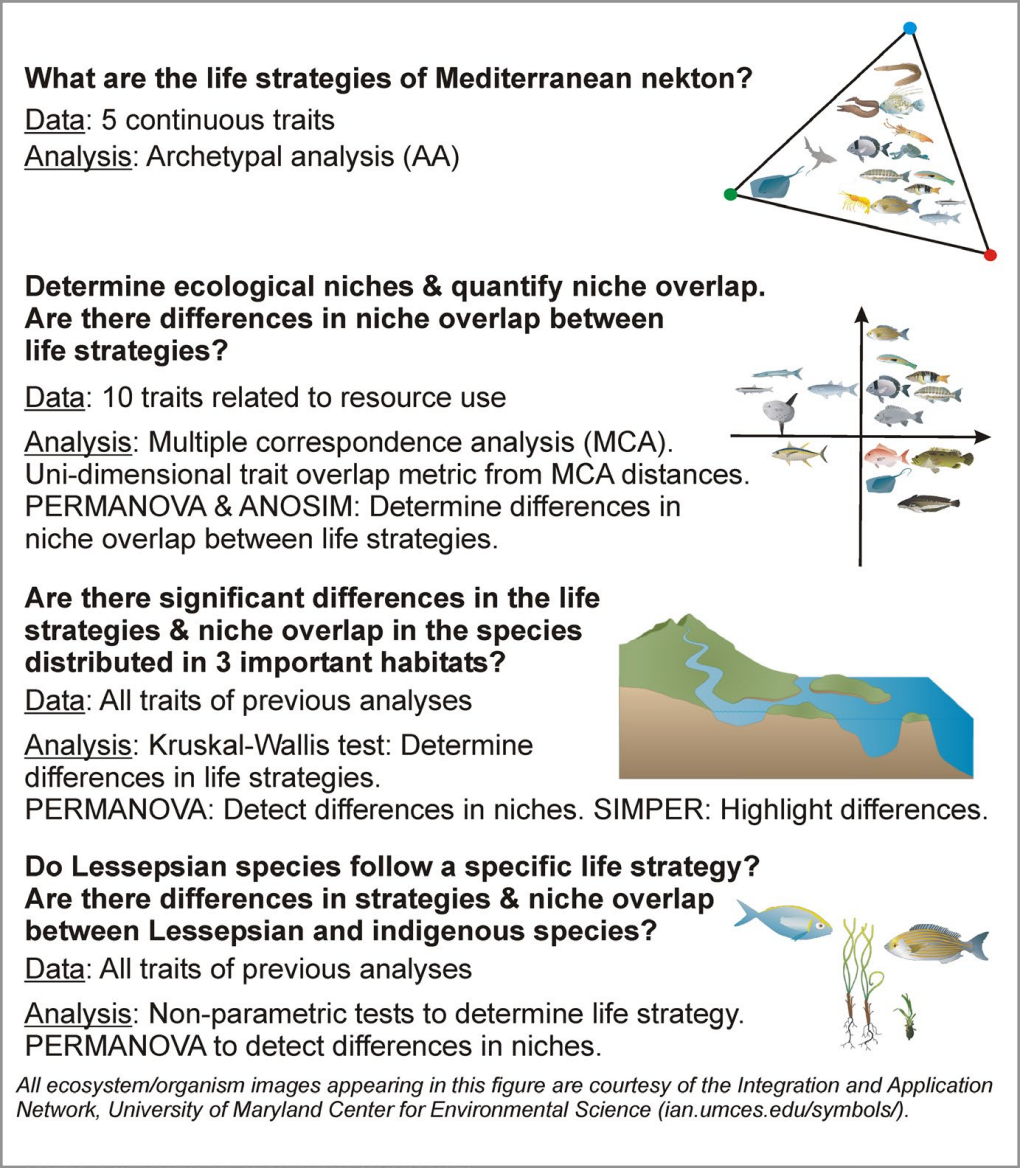
Outline of the aims of the present work, the datasets used and the analyses carried out

The aim of this study is to use biological traits in order to (a) identify the life history strategies of nektonic species of the Mediterranean Sea, (b) define the ecological niches of nektonic species and quantify trait‐based niche overlap concerning specific resource types, and how they are related to life strategies, (c) evaluate the niche overlap and life strategies in the species present in important Mediterranean habitats and investigate whether they are affinities between life strategies and habitats, and (d) determine whether Lessepsian species follow a specific life strategy and examine the level of trait and niche overlap between them and indigenous Mediterranean species (Figure 1).

## 2 MATERIALS AND METHODS

### 2.1 Species selection

We used a subset of a broader dataset of 23 traits of 235 species present in the Mediterranean nekton. Even though the inventory of nektonic species of the Mediterranean Sea is larger, we attempted to include as many species as possible to have a representative depiction of the community, covering pelagic, benthic and benthopelagic species (Tzanatos, Moukas, & Koutsidi, 2020). Special attention was given to include all species found in two Mediterranean habitats known for the biodiversity they host, specifically 66 species found in Posidonia beds (Fernandez, Milazzo, Badalamenti, & D'Anna, 2005; Francour, 1997; Guidetti, 2000; Kalogirou et al., 2010; Moranta, Palmer, Morey, Ruiz, & Morales‐Nin, 2006) and 26 found in coralligène (Ballesteros, 2006). We also included 28 species found in lagoons, as they are highly productive ecosystems (Nicolaidou, Reizopoulou, Koutsoubas, Orfanidis, & Kevrekidis, 2005). Furthermore, a total of 22 Lessepsian species were included (Corsini‐Foka & Economidis, 2007). This dataset accounted for over 98% of the catch biomass of onboard sampling catch composition data in the eastern Ionian Sea (GFCM area: GSA20) in 2013–2014 for all fishing gears (i.e., landings and discards, from the application of the European Union Data Collection Framework—EC 199/2008). On a pan‐Mediterranean scale, this dataset covered ~75% of the species/taxa landed in 2015 according to the FAO‐GFCM Mediterranean‐wide landings dataset (which also includes benthic species).

### 2.2 Traits dataset

The biological traits examined were described by different types of variables: continuous (e.g., maximum life span), range (e.g., optimal depth), and categorical (e.g., spawning habitat: pelagic or benthic). The range type variables were expressed as the average between the minimum and the maximum. Depending on the analysis, the continuous and range type variables were analyzed as continuous or categorical variables. The distribution and range of continuous and range‐type variables were considered for setting the limits for the trait categories (modalities). Furthermore, the trait categories were separated according to their biological significance. Thus, for example, the categories of the trait "optimal depth" were defined as (a) optimal depth 0–50 m (coastal zone, zone of development of benthic photosynthetic organisms), (b) optimal depth 50–200 m (the rest of the continental shelf), and (c) optimal depths >200 m (continental slope/ intercontinental zone). As a result, for the analyses using categorical variables only, 2–6 modalities were created within each trait, in a fashion allowing each species to be included in only one modality per trait (Table 1).

**TABLE 1:** List of traits used in the analysis and categories by trait

Trait	Trait categories (modalities)					
Longevity	1–5 years	5–9 years	10–19 years	>20 years		
Age at maturity^a^	<20%	<40%	<60%			
Fecundity scale^b^	10	100	1,000	10,000	100,000	>100,000
Maximum length	<20 cm	20–50 cm	50–100 cm	>100 cm		
Optimal depth	0–50 m	50–200 m	>200 m			
Depth range	Eurybathic	Stenobathic				
Habitat type	Pelagic	Benthic	Benthopelagic			
Seabed morphology	Open sea	Soft	Hard	Variable		
Trophic level	2	3	4	5		
Diet	Herbivore	Zoobenthivore	Zoobenthivore‐Hyperbenthos	Omnivore	Zooplankton	Piscivore
Feeding behavior^c^	Grazer^c^	Active predator	Ambushing predator			
Spawning period	Winter	Spring	Summer	Autumn	All year	
Spawning habitat	Pelagic	Benthic				

^a^ As a percentage of maximum age.

^b^ Scale of eggs/juveniles per spawn, maximum value indicated.

^c^ Food items have negligible or low mobility related to predator.

The original complete traits dataset (23 traits × 235 species), species selection, and trait categories/modalities definition are also described in more detail in Tzanatos et al. (2020). The complete dataset, together with information on traits as continuous variables, definition of traits categories per species as well as the bibliographic reference for the documentation of each trait per species can be found at https://figshare.com/articles/Koutsidi_Moukas_Tzanatos_23_biological_traits_of_235_species/11347406

From this dataset, we used information on 13 traits (Table 1) that was complete for all the traits of 205 species of nekton (specifically 188 fish, nine cephalopods, and eight crustaceans).

### 2.3 Life strategies

For the identification of ecological niches related to the life history of the species examined, we used Archetypal analysis (Cutler & Breiman, 1994) in a process similar to that followed by Pecuchet et al. (2017). The Archetypal analysis represents each individual in a dataset as a mixture of “individuals of pure type” or “archetypes.” These are a small number of (not necessarily observed) extreme points in a set of multivariate observations; the data are expressed as a probabilistic mixture of archetypes (Cutler & Breiman, 1994; Eugster & Leisch, 2009; Li, Wang, Louviere, & Carson, 2003). As this analysis uses continuous data, five continuous traits of the dataset relevant to life history definition were used: longevity, age at maturity, fecundity, maximum length, and trophic level. Longevity and fecundity were log‐transformed. All five traits were scaled, to ensure equal weights.

As there is no rule for the correct number of archetypes k, we determined its value by running the algorithm for *k* = 1, 2, … 10, by performing 10 iterations and calculating the residual sum of squares (RSS) for each one (Eugster & Leisch, 2009). A small value of k with low RSS was chosen, according to the “elbow criterion.” The R library “archetypes” was used for this analysis (Eugster & Leisch, 2009).

### 2.4 Shared traits, ecological niches, and niche overlap

The identification of species' ecological niches is important in order to determine the role of each species in the environment, and to detect possible competition in case of niche overlap (Pianka, 2006). The concept of competition refers to interaction regarding the exploitation of specific resources, and the traits examined in life strategies are not necessarily relevant to resource use. For this, we used 10 traits relevant to resource exploitation. These traits were divided in three groups based on the type of the main resource use with which these traits can be linked: (a) Maximum length, trophic level, diet and feeding type were used to describe the use of food resources by mature fish, (b) optimal depth, depth range, habitat type, and seabed type were used as a descriptor of habitat use, and (c) spawning period and spawning habitat were considered to reflect spawning habitat use (even though behavioral aspects of some species, for example, carrying their eggs may render the interactions more complex). The two traits: spawning period and spawning habitat can also be relevant for food resource use by larvae and juveniles (as, e.g., nektonic larvae coinciding spatially and temporally can be expected to use the same food resources). Separate species groupings based on sharing the traits relevant to each resource indicated the overlap of simple niches described by the relevant traits. It also indicated trait combinations that were devoid of coverage by any species.

In order to create a more synthetic image and determine traits‐based ecological niches following the “hypervolume” approach (e.g., Pianka, 2006), a multiple correspondence analysis (MCA) was performed on the 10 traits (36 modalities) by 205 species qualitative matrix, using the most important MCA dimension scores of each species. The MCA attempts to detect the underlying structure in a categorical dataset and represent data as points in a low dimensional space; thus, on each MCA dimension, the species modalities became species scores. Furthermore, the MCA classified the species with similar modalities in neighboring locations. As a result, species with similar combinations of traits were placed in adjacent locations in the resulting dimensions and were assumed to have ecological niche overlap and thus similar roles. From the analysis, a total of 26 dimensions explained the total dataset variability. The first four dimensions explained 60.14% (dimension 1:30.27%, dimension 2:12.9%, dimension 3:10.4%, and dimension 4:6.6%) of data variability and were retained as representative of the total, because the percentage of variance explained by each of the remaining dimensions contributed very little to the total dataset variability (similar to Winemiller, Fitzgerald, Bower, & Pianka, 2015). The traits’ contribution to each dimension is presented in the Figure S1.

To estimate the niche overlap between any two species, their coordinates in the four MCA dimensions retained were taken into account. For this, the distances between all possible pairs of 205 species for each of the four retained MCA dimensions were calculated and standardized resulting in four triangular distance matrices. The relevant contribution of each dimension to % variance explained was used to weight the distances of the four triangular matrices (each corresponding to a dimension retained) producing a single weighted distance metric between all species pairs in a final triangular matrix. The smaller this single distance was between two species the higher niche overlap they were considered to have for all resource types. Pairs of species with high distance values were on the contrary considered to have low niche overlap.

To determine whether there are significant differences in the average distances/level of niche overlaps between the different life strategies previously identified, a PERMANOVA (Permutational multivariate analysis of variance) was carried out. As a next step, to determine which groups significantly differed from others, an ANOSIM (Analysis of similarities) on the triangular distance matrix of the MCA dimensions by species was performed. While PERMANOVA can also be designed to indicate pairwise contrasts, ANOSIM was also included, as its R statistic is an absolute measure of the strength of the between‐groups difference, contrary to the PERMANOVA pseudo‐F (Anderson, Gorley, & Clarke, 2008).

Whether niche overlap is high or low must first be measured relative to some null expectation. For this, the library of R, “EcoSimR” (Gotelli, Hart, Ellison, & Hart, 2015) was used to test the overlap in resource use among the set of 205 species (36 modalities of the 10 traits used). The analysis reveals whether the average niche overlap, calculated among all unique pairs of species, is more or less than would be expected if species used resource categories independently of one another. Here, the “pianka” niche overlap index was used, which ranges from 0 (no overlap) to 1 (complete overlap) indicating the mean overlap of all possible species pairs.

### 2.5 Comparisons between species present in important Mediterranean habitats

The archetypal analysis identifies archetypes and provides the percentage that each species is characterized by each archetype. In order to detect whether there are differences between the species assemblages present in important Mediterranean habitats, and possible association(s) with a specific archetype, we compared the arcsine‐transformed percentage scored for each of the three archetypes identified between the groups of species distributed in Posidonia beds, coralligène formations, and lagoons, using the Kruskal–Wallis test, as the parametric prerequisites were not met. In the case that a species appeared in more than one habitat, its score was repeated for all habitats in which it was found.

Furthermore, a PERMANOVA was carried out to detect whether there are differences in the average distances/level of niche overlaps of the species present in Posidonia beds, coralligène formations, and lagoons. Again, as a next step, to determine which groups significantly differed from others, an ANOSIM on the triangular distance matrix of the MCA dimensions by species was performed. Also, to highlight the differences in niche overlap between the species assemblages in Posidonia beds, coralligène formations, and lagoons, a SIMPER (Similarity percentage analysis) on the triangular distance matrix of the MCA dimensions by species was performed.

### 2.6 Comparisons between indigenous and Lessepsian species

In order to detect whether Lessepsian species tend to be associated with a specific archetype, we compared the arcsine‐transformed percentage scored by Lessepsian versus indigenous species for each archetype identified using the Mann–Whitney *U* test (as the parametric prerequisites were not met). Similarly, the distributions were compared using the Kolmogorov–Smirnov test.

Furthermore, a PERMANOVA was performed to detect whether there are differences in average distances/level of niche overlaps between Lessepsian and indigenous species or between archetypes on the triangular distance matrix of the MCA dimensions by species matrix. The independent variable for the first was origin (categorized as Lessepsian and indigenous), and for the latter, it was archetype/life strategy. This was categorized as archetype 1, 2, or 3, intermediate between two archetypes (a species placed between two archetypes but with relatively low contribution of the third one) or intermediate between all three archetypes (species characterized by all archetypes at an almost equal level). Species assigned to an archetype should have a percentage higher than 50% for this archetype, and the percent difference between the first and the second archetypes should be higher than 15%. Species intermediate between two archetypes should have a percent difference between the two highest archetypes lower than 15% and the percentage of the third one lower than 20%. Finally, to assign a species as intermediate between all archetypes the minimum percentage of any archetype should be higher than 20% and the maximum lower than 50%.

As a next step, to determine which groups significantly differed from others, an ANOSIM on the triangular distance matrix of the MCA dimensions by species was performed.

PERMANOVA, ANOSIM, and SIMPER were carried out using the software PRIMER6 (Anderson et al., 2008; Clarke & Gorley, 2006). All inferential tests were carried out at the α = 0.05 significance level.

## 3 RESULTS

### 3.1 Life strategies

**FIGURE 2:**
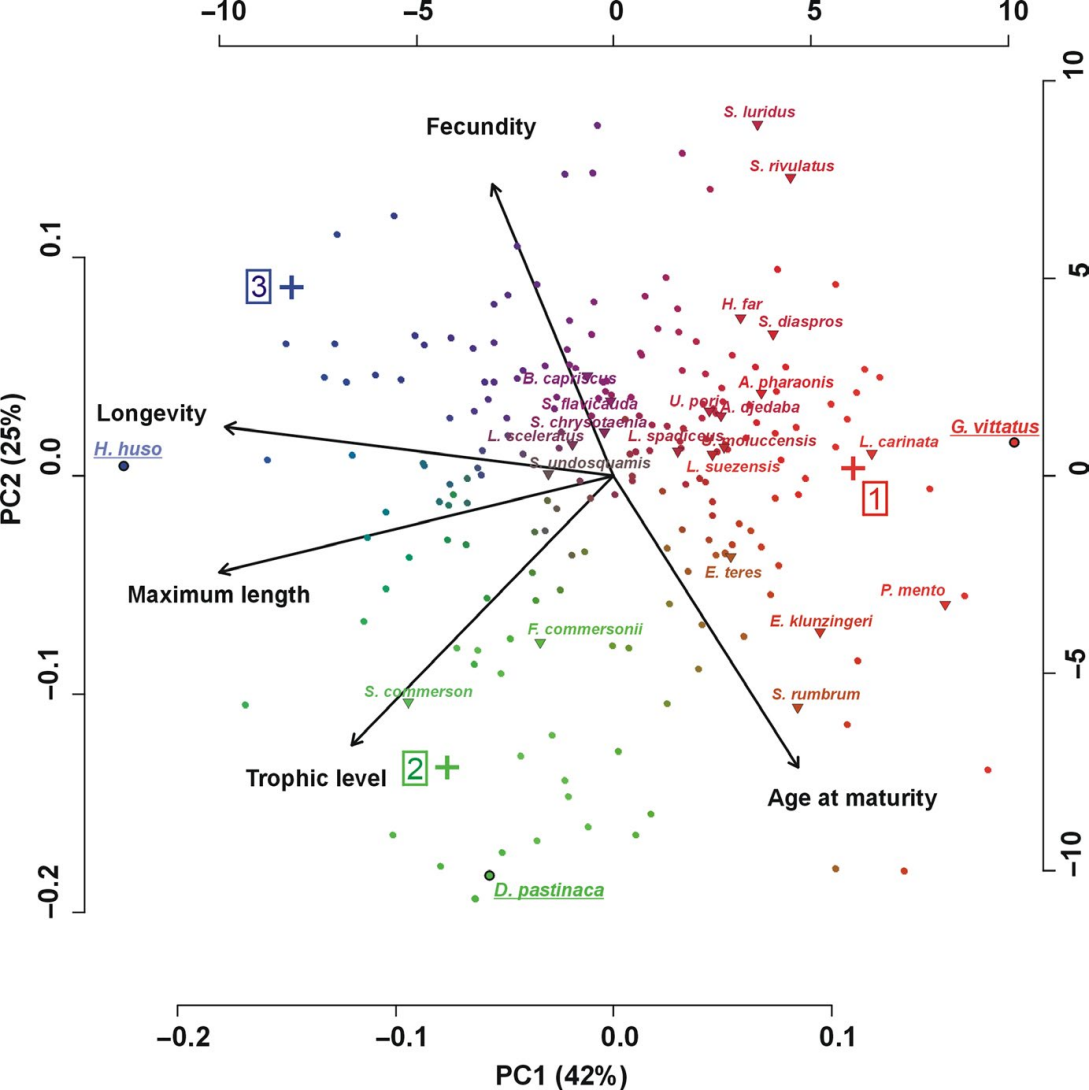
Biplot of the PCA performed on the five traits (longevity, age at maturity, fecundity, maximum length, and trophic level). The resulting archetypes are visualized as colored crosses. Each species is represented by a point, in which the color is a proportional combination of the colors of the three archetypes. Characteristic species of each archetype are underlined. Lessepsian species are represented with colored triangles, and their names are also given

Using the “elbow criterion” (Figure S2), the optimal number of archetypes (niches associated with species life history patterns) was three (Figure 2). An alternative would be to determine five archetypes; however, according to the law of parsimony (also known as “Occam's razor”) that renders more possible the existence of a lower rather than higher number of archetypes, we proceeded with three archetypes.

The representation of the Archetypal analysis results into a principal components analysis (PCA) biplot of the five traits used (Figure 2) which indicated that the first two PCs accounted for more than half of the trait variability. The first PC (~42% of trait variability) was mainly based on longevity, maximum length, and trophic level, while the second (~25%) was mainly based on age at maturity and fecundity. The three archetypes (colored cross in Figure 2) represents three, hypothetical/not existing species in the multivariate set of 205 species. Each possesses a combination of traits that represent a general ecological niche associated with the life history of the species The more similar the color of a species (colored points in Figure 2) is to the color of the archetype, the more its traits are similar to those of the archetype, indicating a shared life history.

The first life history archetype (Figure 2 red cross, archetype‐1) was characterized by traits with a very short life span, small size, low trophic level, relatively late maturation, and low fecundity. Some characteristic species with high similarity to this archetype were, for example, *Pomatoschistus marmoratus*, *Gambusia affinis*, and *Gobius vittatus*. The traits which characterized the second archetype (Figure 2 green cross, archetype‐2) were large size, high trophic level, long life span, late maturation, and low fecundity. Some characteristic species with high similarity with this archetype were, for example, *Centrophorus granulosus*, *Dasyatis pastinaca*, and *Torpedo nobiliana*. The third niche was characterized by species associated with large size, long life span, high fecundity, high trophic level, and early maturation (Figure 2 blue cross, archetype‐3). Some characteristic species for this strategy were, for example, *Polyprion americanus*, *Anguilla anguilla*, and *Huso huso*.

The proportional affinity of each species to the three archetypes identified (Table S1 and Figure S3) indicates that, apart from species that can be clearly associated with a unique archetype, 6.3% of the species occupied intermediate locations between the three archetypes sharing ~30% similarity with each, for example, *Eutrigla gurnardus* sharing 30.0%, 33.5%, and 36.5% similarity with the first, second, and third archetypes, respectively, or *Scorpaena porcus* with the respective percentages being 35.3%, 28.1%, and 36.6%). Additionally, some species (21.4%) occupied locations between two archetypes (mostly between the first and the third archetypes) sharing ~50% similarity with each (e.g., *Chelon labrosus* sharing 47.2% similarity with the first and 52.8% with the third archetype, *Mullus barbatus* with similarities of 56.6% and 43.4%, respectively).

### 3.2 Traits shared, ecological niches, and niche overlap

Traits overlap for specific resources, specifically for (a) habitat use, (b) food resources of adult/mature stages, and (c) spawning habitat use and food of larval/juvenile stages are presented in Table S2. Also, in this table, combinations of traits by resource that are not in use from any of the 205 species analyzed are indicated (empty/possibly available niches). Regarding adult habitat use, eleven trait combinations (out of 36 possible combinations in total, i.e., 30.6%) were empty (not occupied by any species). Concerning spawning habitat, only one (benthic autumn spawning) out of eight possible (12.5%) trait combinations was devoid of any species. Finally, regarding adult stage food resources, 194 trait combinations out of 288 possible (67%) were empty.

**FIGURE 3:**
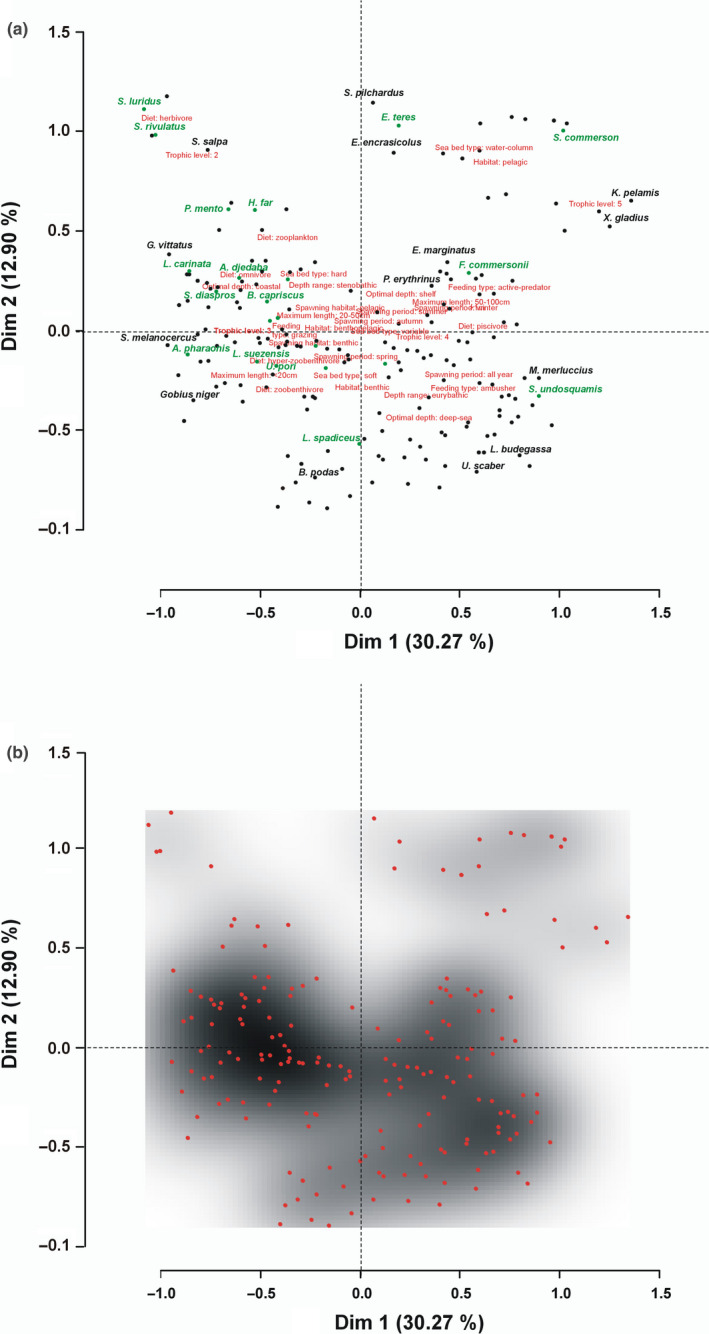
The first two dimensions (Dim 1 and 2, explaining 43.17% of the total variance) derived by the multiple correspondence analysis (MCA) performed on the 10 traits related to resource exploitation for the 205 species examined. (a) The position of the trait categories/modalities in the two‐dimension MCA‐plot is denoted with red font. Lessepsian species and indigenous species are indicated by green and black circles, respectively, with some of the species names given as an example. (b) Density of 205 species in the first two dimensions of MCA. The areas with low or no density indicate relatively empty ecological niches that are possibly available for colonization

Trait‐based ecological niches of the 205 species were derived from the MCA of the 10 traits relevant to resource use. The position of each species on the niche space is indicated by its location/coordinates in the MCA dimensions presented in Figure 3a and in the Supplementary material, Interactive Figure S4. The areas with low or no presence of species points in the niche scheme indicate relatively empty ecological niches that are possibly available for colonization (Figure 3b, indicated by white/nonshaded areas).

**FIGURE 4:**
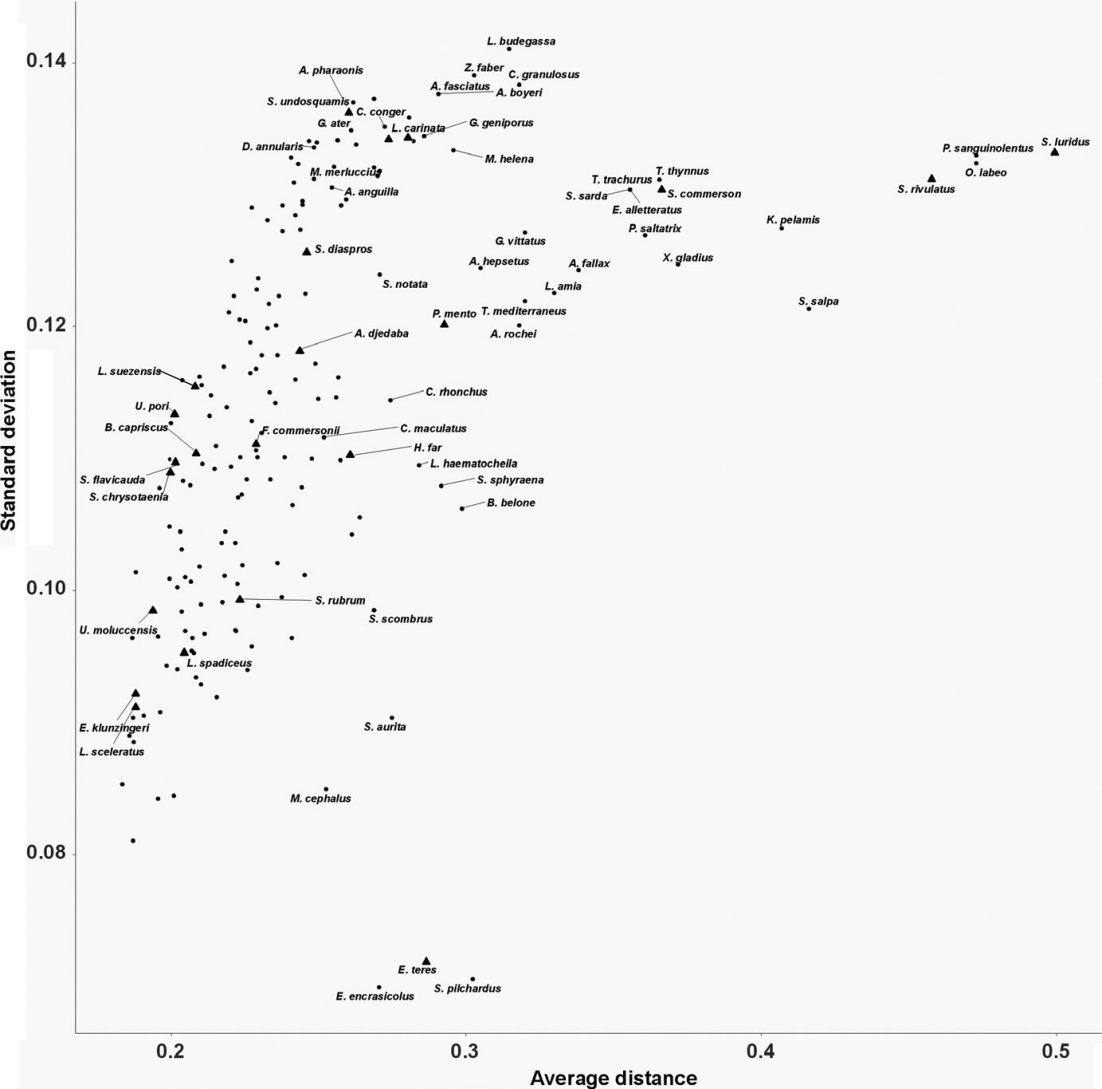
The average distance to the standard deviation of the species examined. A big number of the average distance to standard deviation indicates species that occupied relatively empty ecological niches. Lessepsian species are represented with triangles and their names are also given

Niche overlap was derived from the MCA dimension scores of the species evaluated. Specifically, from the four dimensions retained in the MCA the final single weighted (according to variability explained by each dimension) distance metric (hereafter called “distance”) generated for all pairs of species indicated high overlap in small distances between species pairs (the niche overlap of any species with all others or for specific percentages of overlap is presented in Supplementary material, Interactive Figure S4). Niche overlap ranged from 0% to 74% (the latter being the highest distance found between any two species, note that the range is standardized from 0% to 100% in the Interactive Figure). Pairs of species with zero average distance (15 pairs, e.g., *Atherina boyeri*‐*Aphanius fasciatus*, *Sarda sarda*‐*Euthynnus alletteratus*, *Symphodus mediterraneus*‐*Sparisoma cretense*, and *Synodus saurus*‐*Scorpaena porcus*) in the four MCA dimensions retained had the highest niche overlap. At distances smaller than 10% (i.e., very high niche overlap) about 40% of the species examined had an overlap with 30–55 species (average, *m* = 37 and standard deviation, *SD* = 5.5), for example, *Lithognathus mormyrus* had very high niche overlap with 54 species, *Labrus merula* with 47 and *Mullus barbatus* with 33. At distances ranging between 10% and 20% (considerable niche overlap), 70% of the total species examined were within a cloud of 50–103 species (m = 68, *SD* = 12), for example, *Pagellus erythrinus* within 99 species. On the other hand, very high distances (69%–74%, as mentioned above, no pair of species had a distance higher than 74%) indicated negligible niche overlap, for example, *Merluccius merluccius* with *Siganus luridus* and *Lophius budegassa* with *Parablennius sanguinolentus*. Only a few species had many others at an average distance higher than 60% (*Siganus luridus* with 51 species, *Siganus rivulatus* with 25, *Oedalechilus labeo* with 36, and *Parablennius sanguinolentus* with 34), indicating very low niche overlap. Most of these species were also found to have considerable niche overlap (distance from 10% to 20%) with a small number of others. For example, *Oedalechilus labeo* and *Siganus rivulatus* had five other species placed within this distance, *Sarpa salpa* had six*, Sardina pilchardus* had 10 and *Katsuwonus pelamis* had twelve. The finding that species like *Salpa sarpa*, *Siganus luridus,* and *Katsuwonus pelamis* occupy relatively empty ecological niches (Figure 3a) is also confirmed by their relatively high average distance in relation to its standard deviation (Figure 4).

The PERMANOVA indicated the existence of significant differences in average MCA distances (i.e., level of niche overlaps) between strategies (pseudo‐*F* = 12.573, *p* = .001). The ANOSIM also indicated significant differences between pairwise strategies. Species belonging to archetype‐1 were significantly different in trait composition from all the other strategy levels (Table S1). Archetype‐2 species were significantly different from species intermediate between two or three strategies. Finally, the archetype‐3 species were statistically different from species intermediate between all three strategies.

The null model performed for testing the overlap in resource use among the 205 species using a set of discrete resource categories (trait modalities), indicated higher niche overlap in the original set of species (observed index, mean = 0.477 than that in the random set of species (simulated index, mean = 0.277 ± 7.64E‐07, confidence interval = lower 95%: 0.276, upper 95%: 0.279). Consequently, it can be concluded that niche overlap in the entire species dataset is not random.

### 3.3 Life strategies and ecological niches of species present in important habitats

The archetype affinity scores of species distributed in the three important Mediterranean habitats (Posidonia beds, coralligène, and lagoons) were not found to significantly differ regarding either the first (Kruskal–Wallis, *H* = 3.75, *p* = .153), second (Kruskal–Wallis, *H* = 5.6, *p* = .06), or third (Kruskal–Wallis, *H* = 0.4, *p* = .81) archetype. Thus, it cannot be deduced that one of these habitats is dominated by species following a specific life strategy. Generally, the first (38% of species in Posidonia beds, 35% in coralligène, and 54% in lagoons) and third life history strategies/archetypes (20% of species in Posidonia beds, 31% in coralligène, and 21% in lagoons) are common, while the second strategy is rare (5% of species in Posidonia beds, 4% in coralligène, and 0% in lagoons). Species in intermediate positions between two archetypes are also common over all three habitats (25% of species in Posidonia beds, 19% in coralligène, and 21% in lagoons), but those intermediate between all three are common in Posidonia and coralligène (12% of species in either) and rather rare in lagoons (5%).

The PERMANOVA indicated significant differences in the average MCA‐weighted distances (niche overlap) of the species distributed between habitats (pseudo‐*F* = 3.8723, *p* = .008). The ANOSIM also indicated significant differences in the MCA dimensions of the species between pairs of habitats: Species appearing in lagoons were significantly different in trait composition from those of coralligène assemblages (R statistic = 0.053, *p* = .042), albeit the relationship was weak. SIMPER analysis indicated an average dissimilarity 58.43% between lagoons and coralligène formations. The modalities responsible for the dissimilarity for the species distributed in lagoons were as follows: trophic level: 3, optimal depth: 0–50 m, sea bed type: soft, spawning habitat: pelagic, and spawning period: summer and spring, while the modalities that were responsible for the dissimilarity for the species over coralligène formations were as follows: trophic level: 4, all ranges of optimal depth, sea bed type: variable, spawning habitat: pelagic and benthic, and spawning period: summer.

**FIGURE 5:**
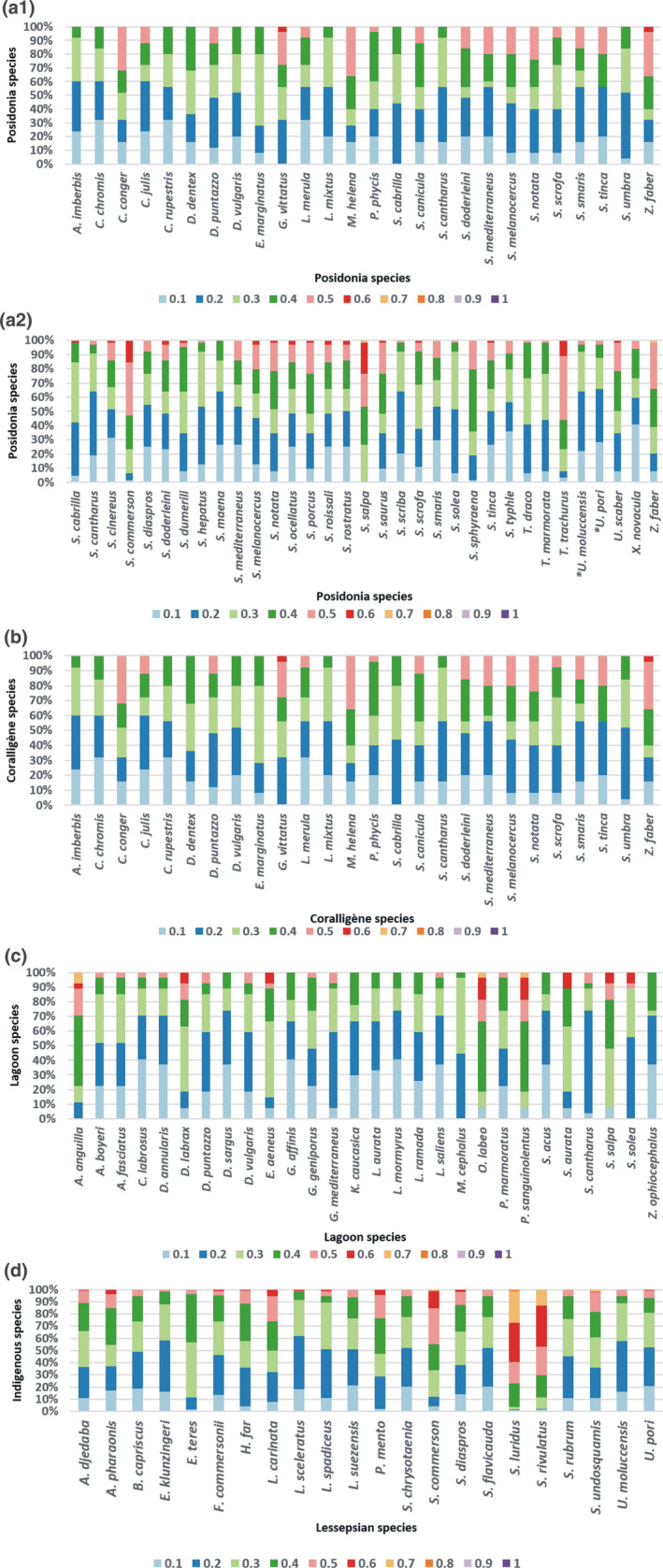
Frequencies of species distributed in Posidonia beds (a1 and a2), coralligène formations (b), and lagoons (c) according to the pairs of distances with the other species of the same habitat based on the 10 traits used. (d) Frequencies of the average distances of the Lessepsian species according to the pairs of distances with all the indigenous species of the dataset based on the 10 traits used. The smaller the distance was between two species, the highest niche overlap they were considered to have and thus the highest competition for all resource types

Examining the niche overlaps between species within the assemblage of each habitat, concerning the 65 species present in Posidonia beds, most of them were within a small distance (lower than 30%), that is, had high niche overlap with at least 70% of the other species found there (Figure 5a1,a2). However, there were two exceptions indicating low niche overlap and overall trait overlap with the other species in Posidonia beds: *Scomberomorus commerson* had an average distance of 0%–40% with half of the other species in Posidonia beds and with the other half a distance higher than 40%. *Sarpa salpa* had a distance 50%–60% with at least 50% of the other species in Posidonia beds (Figure 4). Concerning the 26 species present in coralligène formations, most of them were within a small distance (<40%) with at least 80% of the other species found there (Figure 5b). There were four exceptions indicating low niche overlap with other species found in coralligène: *Conger conger*, *Gobius vittatus*, *Muraena helena,* and *Zeus faber* all had an average distance lower than 50% (high overlap) with half of the other species in coralligène and with the other half a distance higher than 50% (low overlap). Concerning the 28 species present in lagoons, most of them were within a distance lower than 30% (high niche overlap) with at least 70% of the other species in lagoons (Figure 5c). However, there were three exceptions indicating low niche overlap and overall trait overlap with the other species in lagoons: *Anguilla anguilla*, *Oedalechilus labeo,* and *Parablennius sanguinolentus* had an average distance of 50%–70% with the 30% of the other species in lagoons.

### 3.4 Life strategies and ecological niches of Lessepsian species

Regarding life strategies, the 22 Lessepsian species were found to significantly differ from indigenous species in the median value of the first archetype (Mann–Whitney, *W* = 796.0, *p* = .002) where they attained significantly higher values [*μ*(indigenous) = 0.27, *μ*(Lessepsian) = 0.39]. On the contrary, Lessepsian and indigenous species did not demonstrate significant differences regarding neither the second (Mann–Whitney, *W* = −410.5, *p* = .113), nor the third archetype (Mann–Whitney, *W* = −354.5, *p* = .178). The distributions were also significantly different between the indigenous and Lessepsian species for the first and the second archetypes (*D* = 0.339, *p* = .021 and *D* = 0.409, *p* = .02, respectively, in the latter case the Lessepsian species obtained lower frequencies than indigenous ones), but not for the third (*D* = 0.300, *p* = .057). Thus, overall, Lessepsian species can be clearly associated with the first archetype (also indicated in Figure 2 and in Table S1).

Concerning the Lessepsian species examined, most of them were within a distance ranging from 0% to 40% with most (>70%) of the indigenous ones (Figures 4 and 5d). However, there were three exceptions indicating low niche overlap with indigenous species: *Scomberomorus commerson* had an average distance of 0%–40% with half of the indigenous species and with the other half a distance higher than 40%. *Siganus luridus* and *Siganus rivulatus* had a distance higher than 50% with at least 70% of the indigenous species. Overall, however, comparing the average niche overlaps of the Lessepsian species against those of the indigenous ones did not indicate significant differences between these two origin groups (pseudo‐*F* = 1.0174, *p* = .355).

## 4 DISCUSSION

In the present study, we analyze five life cycle biological traits to detect the life history strategies of 205 Mediterranean nektonic species. We document three life history strategies. Using 10 biological traits related to resource exploitation, we define ecological niches and detect niche and trait overlap (both overall and concerning specific resource types), generating a unique distance metric to assess niche overlap between all species pairs. Taking a closer look at Lessepsian species, we document their affinity to the first life strategy and examine niche overlap between them and indigenous species indicating, for example, the relatively empty ecological niche occupied by the herbivorous invaders *Siganus luridus* and *S. rivulatus*. Furthermore, we document that most species occurring in important habitats have a relatively high niche overlap with species occurring in the same habitat (Figure 5).

As stated above, we identify three main life history niches of Mediterranean nekton. Even though in this study, we do not use the same traits as the theoretical framework for the detection of life history strategies (Pecuchet et al., 2017; Winemiller & Rose, 1992)—as there is absence of the traits “parental care” and “offspring size” in our dataset—the species characteristics occupying the three detected life history niches documented here (red, green, and blue in Figure 2) are similar to the characteristics of the species following the life history strategies defined there: opportunistic, equilibrium, and periodic. The species following the first life history niche correspond to the opportunistic strategy. They have a short generation time, thus show a high intrinsic growth rate, despite their relatively low individual fecundity (King & McFarlane, 2003). Additionally, they have high natural mortality (Beverton & Holt, 1959). The group of species in this strategy comprises mainly small pelagic or coastal, herbivorous benthopelagic species associated with habitats defined by disturbance and high variability, but also with high energy resources (King & McFarlane, 2003). Most Lessepsian species are associated with this strategy in our work.

The species of the second life history niche are similar to the equilibrium strategy of Winemiller and Rose (1992). There are mostly chondrichthyans, are characterized by late maturation (extended brooding season increases the risk of mortality before reproduction) and low fecundity (low dispersal), traits that make them vulnerable to environmental changes (Stevens, Bonfil, Dulvy, & Walker, 2000) and human impacts (Lucifora, García, Menni, & Worm, 2012). Thus, they are favored in stable habitats, with low environmental changes (Mims & Olden, 2012). They have also been documented to show low resilience to fishing mortality and low recovery rates after population decline (Hoenig & Gruber, 1990; Stevens et al., 2000). Most chondrichthyans are widespread marine food chain top predators. Because of their rare traits combinations (the rarity of low fecundity has been documented by Koutsidi, Tzanatos, Machias, & Vassilopoulou, 2016 and Tzanatos et al., 2020), their removal could result in species replacement and niche vacancy, potentially influencing ecosystem structure and functioning (Gouraguine et al., 2011).

The third life history niche is similar to the periodic strategy (Pecuchet et al., 2017; Winemiller & Rose, 1992) and included large bodied species with long life span and high fecundity. Species with these traits (e.g., *Polyprion americanus* and *Pseudocaranx dentex*) are able to cope with variable and unpredictable environments by producing large numbers of eggs (Hutchings, 2002). Thus, this niche includes species likely to be favored in highly seasonal environments (Mims & Olden, 2012). High longevity and fecundity enhance these species’ survivorship though low productivity regimes and storage energy for future more favorable environments (King, McFarlane, & Beamish, 2001). It is important to note that according to our findings (Table S1), this niche is occupied by several species of high commercial value and targeted by fisheries like representatives of the family Sparidae (e.g., *Sparus aurata* and *Dentex dentex*) or other commercial species (*Anguilla anguilla* and *Epinephelus* spp.).

In our results, several species are placed in intermediate locations between the three life strategy archetypes. Similar to our findings, King and McFarlane (2003) suggest the existence of an intermediate strategy, as the life histories of most species are not clear. Several species targeted and discarded by fisheries are found to follow this intermediate life strategy, for example, *Sardina pilchardus* (commercial) and *Diplodus annularis* (typical bycatch of various Mediterranean fisheries), respectively.

Regarding the overlap in both trait groups related to a specific resource independently (adult food, adult habitat, and spawning habitat) and in the ecological niches described by combining all 10 traits, it is important to note that some combinations of traits/niches may be empty because no species with these trait combinations is distributed in the study area (or was not included in the dataset analyzed). However, there is also the possibility that the specific traits set does not exist, as it is known that traits can exist in specific combinations following the main life strategies (e.g., King & McFarlane, 2003 and also documented here); thus, for example, combinations of small size with high trophic level are not found because they are not possible, not because the actual niche is vacant.

Many species have relatively small distance from others, and thus, there is relatively high niche overlap between these two species. The examination of the distances, not on a pairwise level, but for the entire assemblage, can be an indication of community assembly rules through competitive interactions. This interpopulation process may eventually result in combinations of species that will continue to coexist (McGill, Enquist, Weiher, & Westoby, 2006). However, further insights on the community assembly rules would need to use actual abundance (and not presence only) data as this would allow the quantification of interspecific and intraspecific competition. Furthermore, it should be noted that here we evaluate niche overlap. This overlap may be linked to potential competition based on the availability of the relevant resource (e.g., food for adult stages). However, for actual interspecific competition to exist, the different species need to co‐occur in the same habitat. Even if two species co‐occur and group together (regarding the use of a specific resource), this does not necessarily reflect competition. Resource abundance has also to be taken into account since these species could be exploiting a very common, plentiful food resource (Brocksen, Davis, & Warren, 1968).

Regarding specific habitats (Posidonia beds, coralligène, and lagoons), more species with a high niche overlap with others were found to exist in Posidonia beds (but the number of species occurring there is anyway higher). Species distributed in these important Mediterranean habitats have no significant affinity with a specific life history strategy, as generally the opportunistic and periodic strategies are common and the equilibrium strategy is rare in all three habitats while “intermediate” species are also generally common. This is an interesting finding and may indicate that there is no environmental filtering tending to select only one strategy to prevail over a specific habitat, but the communities are composed of species whose fitness can be attained by a combination of different adaptations, that is, following different strategies (McCann, 1998). Even highly dynamic environments showing intense seasonality, like lagoons, host both species with an affinity to the periodic strategy (e.g., *Anguilla anguilla, Dicentrarchus labrax, and Sparus aurata*) and opportunistic species (e.g., *Gambusia affinis*, *Gobius geniporus, and Zosterisessor ophiocephalus*). The diversity in traits (and strategies followed) could be a factor that contributes to the stability of these communities in the long term, as it can generate multiple responses on various spatio‐temporal scales to different stressors (Statzner & Beche, 2010), however, to investigate this would require abundance data and probably a long time series. Independently of strategy, however, the species appearing in lagoons were different (but not strongly) in trait composition from these of coralligène assemblages. Lagoons are characterized by species distributed in shallow waters with low trophic level, for example *Chelon labrosus*, *Diplodus annularis,* and *Liza aurata*, in contrast with coralligène assemblages that characterized by species distributed in deeper waters with higher trophic level (e.g., *Conger conger*, *Muraena helena*, and *Scorpaena notata*). Most traits related to the contrasts between these two habitats (apart from trophic level) are naturally related to their characteristics, for example depth and seabed type (Ballesteros, 2006; Nicolaidou et al., 2005), thus indicating some environmental filtering on traits related to habitat properties.

Concerning Lessepsian species, the lack of significant difference between them and indigenous ones in their average distance from all other species is complemented by the finding that most have high niche overlap with many native species (PERMANOVA results and Figure 5d). Arndt, Givan, Edelist, Sonin, and Belmaker (2018) document that competitive exclusion between Lessepsian and native populations does not explain the decline of native populations, despite the possession of similar traits. Also, they found that the exclusion by competition is only possible in the case of *Mullus barbatus* and *Mullus surmuletus*, caused by species of the genus *Upeneus*. In the present study, *Upeneus moluccencis* and *Upeneus pori* have a very high niche overlap with *Mullus barbatus* and *Mullus surmuletus* (the distance between them being smaller than 1%). *Siganus luridus* and *Siganus rivulatus* have high niche overlap with only a few indigenous species: *Sarpa salpa* with both and *Parablenius sanguinolentus* with *Siganus rivulatus*. Competition between the two species of the genus *Siganus* with *Sarpa salpa* has been previously documented by Bariche, Letourneur, and Harmelin‐Vivien (2004), Giakoumi (2014). Lundberg and Golani (1995), and Sala, Kizilkaya, Yildirim, and Ballesteros (2011) report that the successful spread and establishment of *Siganus* sp. is due to the low number of rival species (few herbivores), that leads to the existence of free niches to occupy. Our finding that *Siganus* spp. have small niche overlap with most indigenous species (Figures 4 and 5d) probably explains their establishment due to their ability to occupy underexploited or vacant niches (Mavruk & Avsar, 2008; Por, 1978).

As mentioned above, in the present work most Lessepsian species are related to the first life strategy. This possibly makes these species capable of adapting to environments characterized by variability (King & McFarlane, 2003) and could be key for their spread and establishment in the Mediterranean, as the ability to adapt to various environmental conditions is generally accepted as a precondition for successful establishment of invaders in marine habitats (Moyle & Marchetti, 2006; Safriel & Ritte, 1980).

Violle et al. (2017) point out that functional rarity has an important role in ecosystem processes. Species with high niche overlap with only a small number of other species (i.e., with only a few other species in neighboring distances (Figure S4) could be considered as species that serve unique ecological roles within the ecosystem and occupy relatively empty ecological niches (e.g., *Engraulis encrasicolus* and *Sarpa salpa*). Thus, rare functions could be served by species with rare traits. Koutsidi et al. (2016) detect commercial fish species with rare combinations of traits, like *Engraulis encrasicolus,* that are also documented here as a species with low niche overlap and indicate rare modalities (possessed by a few species) like spawning in autumn. Also, Tzanatos et al. (2020) document rare modalities like winter spawning, planktivorous diet, hard seabed type, and low fecundity. Species possessing rare trait combinations or rare modalities may be keystone species, that is, species whose relative importance for the community is much greater than their relative abundance (Bond, 2001). The decline, or in the extreme case, extirpation of their populations, may deprive the ecosystem from ecological functions and lead to the creation of vacant niches. In general, the existence of vacant niches (nonshaded areas in Figure 3b, but also see Figure 4, Figure S4) in the assemblage could potentially increase the possibility of the spread and establishment of invading populations (Belmaker, Parravicini, & Kulbicki, 2014; Givan et al., 2017). Here, herbivory was not only documented to be shared by only a few species, but also two of them are the Lessepsians of the genus *Siganus* that have high niche overlap with the indigenous *Sarpa salpa*. Furthermore, the rarity of herbivory has previously been documented in the Mediterranean Sea and North Atlanic/Northeast Pacific by Beukhof, Dencker, Palomares, and Maureaud (2019) and Tzanatos et al. (2020), respectively. Herbivory is a crucial aspect of ecosystem functioning as changes in herbivory rates may cause loss of habitat formers or replacement by others (Vergés et al., 2014); thus, replacement of the indigenous species by the invading ones could alter coastal ecosystems.

The present study used functional traits of 205 species of Mediterranean nekton, and although these species constitute a major part of fisheries catches, they do not reflect the entire biodiversity of Mediterranean fish assemblages. However, the species not examined in this work generally appear in low abundances in the ecosystem or, generally, the information regarding their biological traits is incomplete. Unfortunately, no overall assessment of species abundance in the nekton community exists. Fisheries, because of the existence of target species for each fishing gear, tend to select certain species. The most “objective” estimates of abundance come from trawl surveys (e.g., International Bottom Trawl Survey‐IBTS, Mediterranean International Trawl Survey‐MEDITS) as the trawl gear sweeps the seabed thus providing an objective estimate of density per area. Still, these surveys are known to underestimate pelagic species and species preferring hard substrates. The only other source of information is stock assessments; however, these are only carried out for only a handful of species in the Mediterranean Sea. Another limitation of the present study is related to the fact that here we categorize each species in only one trait (i.e., not taking into account the intraspecific trait variability). Finally, there is widespread discussion about the optimal number of traits to use (Pecuchet et al., 2017); thus, perhaps our findings would be different if we used a different number of traits. This is also the reason for selecting only traits that can be clearly associated with resource use and also distinguishing different resource uses in analyses related to species roles and competition.

Fishing gears can be associated with specific traits, selecting species that possess them and thus decreasing the trait presence in the community (Koutsidi et al., 2016; Mbaru, Graham, McClanahan, & Cinner, 2020). Naturally, the exploitation of stocks through fisheries has direct (by removing the catches of some species) and indirect (by changing predation and competition patterns due to the relative change in the abundance of different populations in the community) impacts on the community composition. This may in turn have significant implications for the traits composition and the associated functioning at the community level and could be examined in future modeling simulations.

The findings of the present work could extend to study community assembly rules using objective abundance data (e.g., survey data) in the future. Additionally, the niche overlap documented here could help establish an understanding of species population dynamics and be used in fisheries management (King & McFarlane, 2003), for example, by incorporating into fisheries models the dynamics of species competing with the stock under examination (as, e.g., sometimes the “gap” left by the reduction in the abundance of target species can be filled by competing unexploited ones). Especially within the framework of the Ecosystem Approach to Fisheries (e.g., Jennings & Rice, 2011), the dynamics of entire groups of species sharing the same life strategies could be examined. Furthermore, as species sharing life strategies may be expected to react to exploitation (or other stressors, environmental or anthropogenic) in similar ways, responses of populations documented in the past can be predicted to occur in data‐poor species under similar stressors or regimes, informing on the possible effects of management on species not evaluated in stock assessments. At a further step, this approach could be used to predict the potential responses of populations in the rapidly changing environment, taking into account fisheries or other stressors of nektonic assemblages. Finally, combining trait composition with fish abundance dynamics could be helpful in quantifying the taxonomic and functional resilience of marine ecosystems to anthropogenic change.

## CONFLICT OF INTEREST

The authors declare no conflict of interest.

## AUTHOR CONTRIBUTION

**Martha Koutsidi:** Conceptualization (equal); Data curation (lead); Formal analysis (lead); Methodology (lead); Writing‐original draft (lead); Writing‐review & editing (lead). **Catherine Moukas:** Data curation (supporting); Formal analysis (supporting); Methodology (supporting). **Evangelos Tzanatos:** Conceptualization (equal); Data curation (supporting); Formal analysis (supporting); Funding acquisition (lead); Methodology (supporting); Supervision (lead); Writing‐original draft (supporting); Writing‐review & editing (supporting).

## Supporting information

Appendix S1

## Data Availability

Data are available from figshare (Koutsidi, Moukas, & Tzanatos, 2018) at: https://figshare.com/articles/Koutsidi_Moukas_Tzanatos_23_biological_traits_of_235_species/11347406
