# Supplementary material for: Trait‐based life strategies, ecological niches, and niche overlap in the nekton of the data‐poor Mediterranean Sea
Source: Ecol Evol. 2020 May 26;10(14):7129–44. doi: 10.1002/ece3.6414 (PMC7391318; doi:10.1002/ece3.6414)
Supplement: Supplementary file 1 — Appendix S1 [file ECE3-10-7129-s001.docx]

**Appendix S1**

**
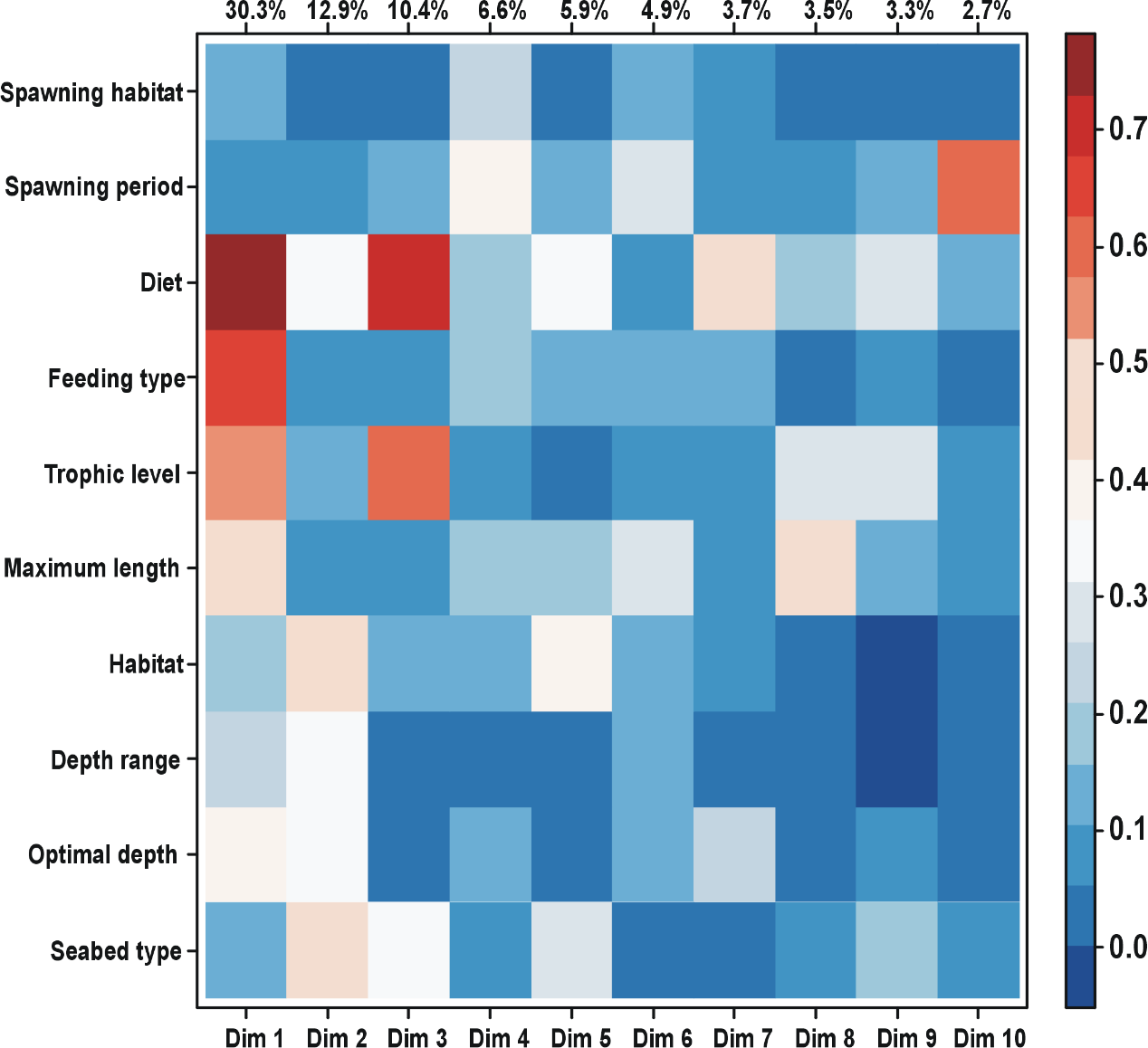
**

Figure S1: The contribution of each of the ten traits to each dimension. The first four dimensions explained 60.14 % of data variability and were retained as representative of the total.

**
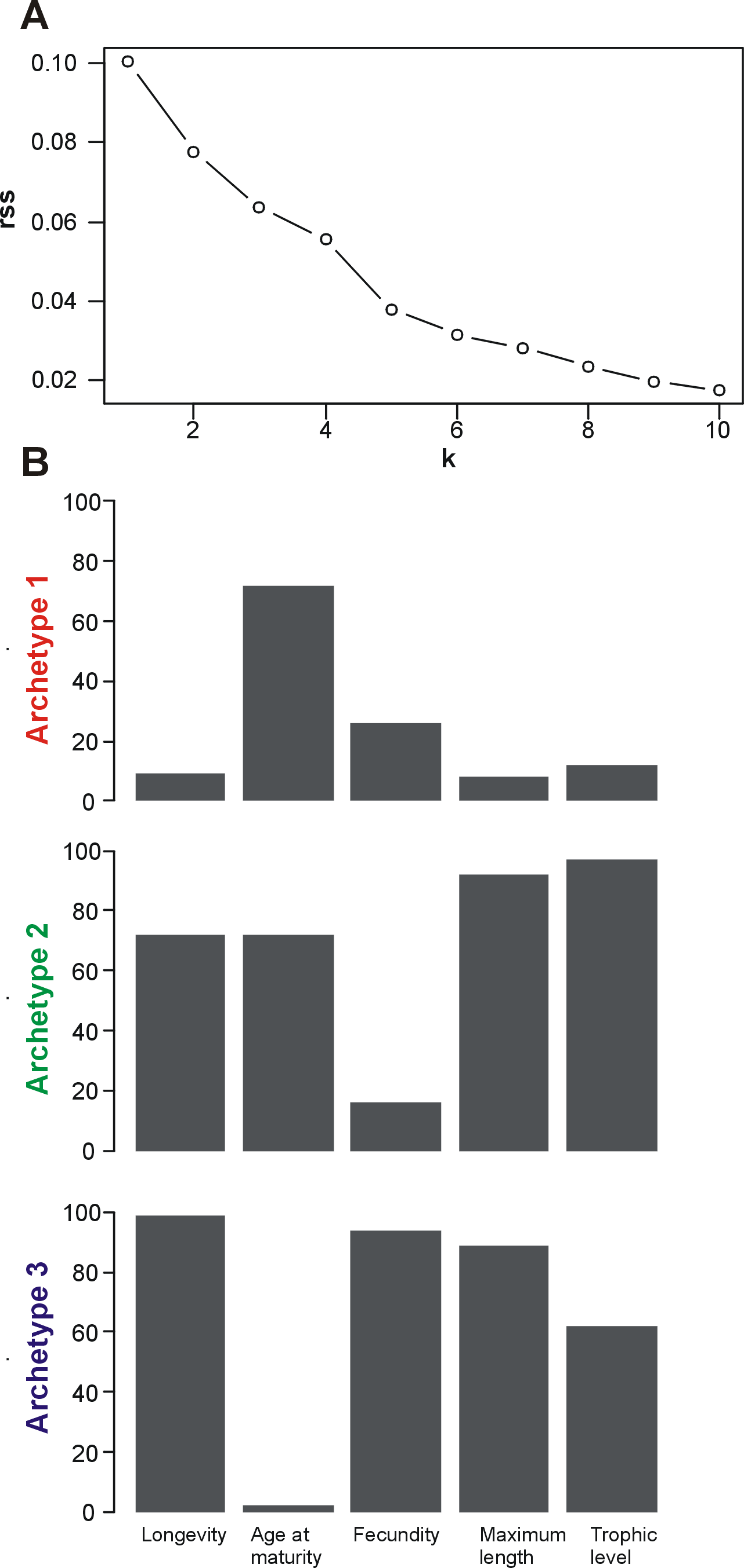
**

Figure S2: A: Calculation of residual sum of squares (RSS), by performing 10 iterations for k= 1,2, …, 10 (k: number of archetypes). The “flattening” of the curve indicates the correct value of k, according to the “elbow criterion”. B: Barplot visualizing the percentiles of the traits for each archetype.


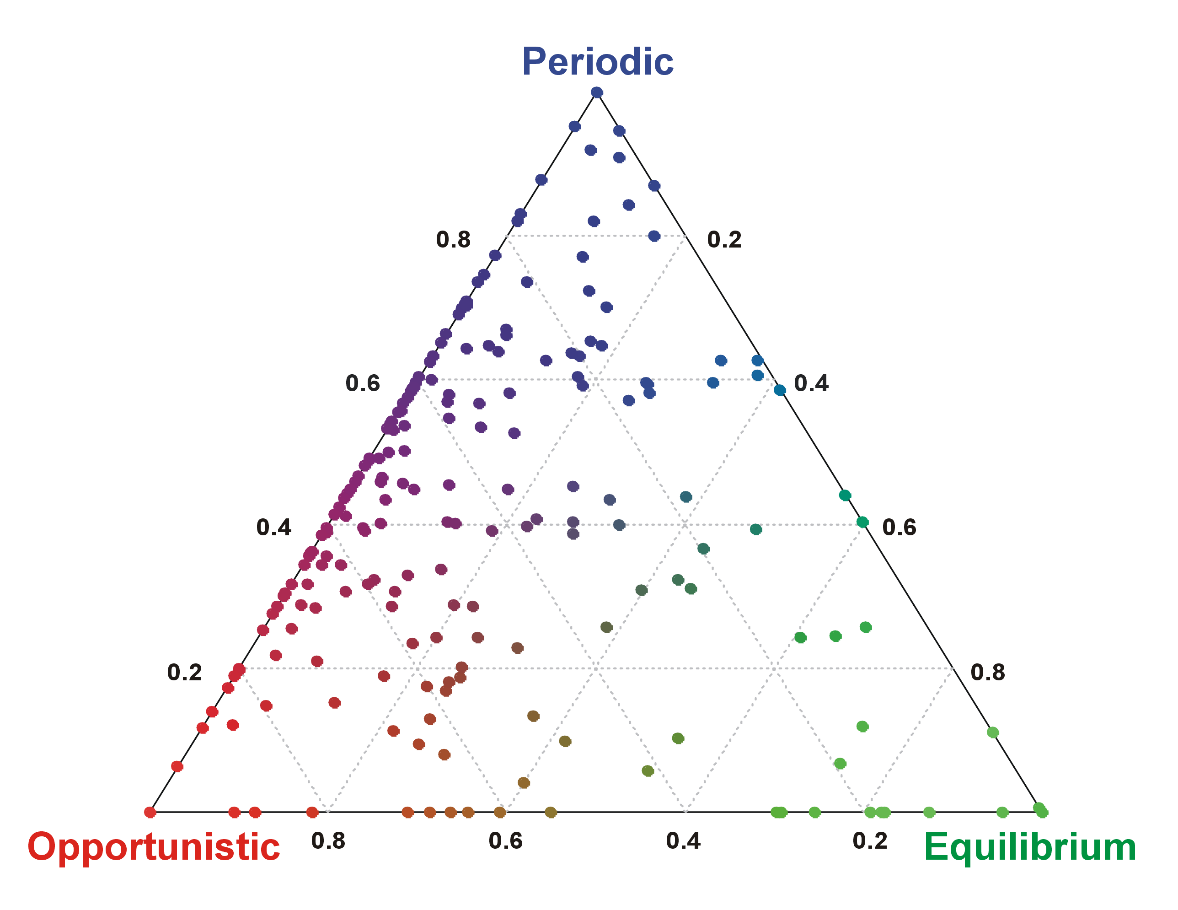


Figure S3: The position of each of the species in the dataset concerning each strategy. Opportunistic (Archetype 1), Equilibrium (Archetype 2), Periodic (Archetype 3).

Interactive Figure S4: Niche overlap based on the average distances between the 205 species examined, based on the MCA performed on the ten resource traits. The smaller this distance is between two species the highest niche overlap they are considered to have for all resource types. Available at: <https://competition.shinyapps.io/Interactive_Figure_S4/>

Table S1: Table of the percentage that each species is characterized by each of the three archetypes. A1 : Archetype 1, A2 : Archetype 2, A3 : Archetype 3, O: Opportunistic, E: Equilibrium, P: Periodic, Or: Origin (Lessepsian: L or Indigenous: I).

| **species** | **A1 (O)** | **A2 (E)** | **A3 (P)** | **Or** | **species** | **A1 (O)** | **A2 (E)** | **A3 (P)** | **Or** |
| --- | --- | --- | --- | --- | --- | --- | --- | --- | --- |
| *Acipenser gueldenstaedtii* | 0.099 | 0.070 | 0.831 | I | *Dentex gibbosus* | 0.265 | 0.030 | 0.705 | I |
| *Acipenser stellatus* | 0.185 | 0.065 | 0.750 | I | *Dentex macrophthalmus* | 0.316 | 0.041 | 0.643 | I |
| *Acipenser sturio* | 0.148 | 0.044 | 0.808 | I | *Dentex maroccanus* | 0.527 | 0.061 | 0.412 | I |
| *Alepes djedaba* | 0.654 | 0.000 | 0.346 | L | *Dicentrarchus labrax* | 0.339 | 0.000 | 0.661 | I |
| *Alosa fallax* | 0.350 | 0.106 | 0.545 | I | *Diplodus annularis* | 0.507 | 0.000 | 0.493 | I |
| *Anguilla anguilla* | 0.106 | 0.000 | 0.894 | I | *Diplodus puntazzo* | 0.401 | 0.000 | 0.599 | I |
| *Aphanius fasciatus* | 0.904 | 0.004 | 0.092 | I | *Diplodus sargus* | 0.484 | 0.141 | 0.375 | I |
| *Apogon imberbis* | 0.602 | 0.066 | 0.332 | I | *Diplodus vulgaris* | 0.404 | 0.227 | 0.369 | I |
| *Apogonichthyoides pharaonis* | 0.735 | 0.000 | 0.265 | L | *Eledone cirrhosa* | 0.643 | 0.357 | 0.000 | I |
| *Argentina sphyraena* | 0.592 | 0.269 | 0.139 | I | *Eledone moschata* | 0.696 | 0.304 | 0.000 | I |
| *Aristaeomorpha foliacea* | 0.599 | 0.057 | 0.344 | I | *Enchelycore anatina* | 0.081 | 0.301 | 0.617 | I |
| *Aristeus antennatus* | 0.564 | 0.000 | 0.436 | I | *Engraulis encrasicolus* | 0.685 | 0.000 | 0.315 | I |
| *Arnoglossus laterna* | 0.645 | 0.022 | 0.333 | I | *Epinephelus aeneus* | 0.211 | 0.156 | 0.633 | I |
| *Atherina boyeri* | 0.814 | 0.093 | 0.094 | I | *Epinephelus alexandrinus* | 0.256 | 0.154 | 0.590 | I |
| *Atherina hepsetus* | 0.733 | 0.165 | 0.102 | I | *Epinephelus caninus* | 0.110 | 0.000 | 0.890 | I |
| *Auxis rochei* | 0.366 | 0.397 | 0.237 | I | *Epinephelus marginatus* | 0.086 | 0.026 | 0.888 | I |
| *Balistes capriscus* | 0.469 | 0.000 | 0.531 | L | *Equulites klunzingeri* | 0.674 | 0.326 | 0.000 | L |
| *Belone belone* | 0.211 | 0.442 | 0.347 | I | *Etmopterus spinax* | 0.268 | 0.732 | 0.000 | I |
| *Boops boops* | 0.634 | 0.000 | 0.366 | I | *Etrumeus teres* | 0.569 | 0.280 | 0.150 | L |
| *Bothus podas* | 0.645 | 0.056 | 0.299 | I | *Euthynnus alletteratus* | 0.338 | 0.055 | 0.607 | I |
| *Callionymus lyra* | 0.615 | 0.250 | 0.135 | I | *Eutrigla gurnardus* | 0.326 | 0.239 | 0.435 | I |
| *Callionymus maculatus* | 0.743 | 0.064 | 0.193 | I | *Farfantepenaeus aztecus* | 0.747 | 0.000 | 0.253 | I |
| *Callionymus risso* | 0.848 | 0.000 | 0.152 | I | *Fistularia commersonii* | 0.247 | 0.461 | 0.292 | L |
| *Caranx rhonchus* | 0.414 | 0.000 | 0.586 | I | *Gaidropsarus mediterraneus* | 0.533 | 0.205 | 0.262 | I |
| *Centracanthus cirrus* | 0.657 | 0.091 | 0.252 | I | *Galeus melastomus* | 0.149 | 0.623 | 0.228 | I |
| *Centrophorus granulosus* | 0.000 | 0.970 | 0.030 | I | *Gambusia affinis* | 0.936 | 0.000 | 0.064 | I |
| *Chelidonichthys cuculus* | 0.525 | 0.263 | 0.212 | I | *Gobius ater* | 0.871 | 0.000 | 0.129 | I |
| *Chelon labrosus* | 0.472 | 0.000 | 0.528 | I | *Gobius geniporus* | 0.733 | 0.119 | 0.148 | I |
| *Chlorophthalmus agassizii* | 0.586 | 0.271 | 0.143 | I | *Gobius niger* | 0.719 | 0.055 | 0.226 | I |
| *Chromis chromis* | 0.610 | 0.149 | 0.241 | I | *Gobius vittatus* | 1.000 | 0.000 | 0.000 | I |
| *Citharus linguatula* | 0.520 | 0.084 | 0.396 | I | *Helicolenus dactylopterus* | 0.318 | 0.000 | 0.682 | I |
| *Conger conger* | 0.042 | 0.342 | 0.615 | I | *Hemiramphus far* | 0.689 | 0.000 | 0.311 | L |
| *Coris julis* | 0.614 | 0.000 | 0.386 | I | *Hexanchus griseus* | 0.000 | 0.554 | 0.446 | I |
| *Coryphaena hippurus* | 0.225 | 0.152 | 0.623 | I | *Hippocampus guttulatus* | 0.630 | 0.114 | 0.255 | I |
| *Ctenolabrus rupestris* | 0.599 | 0.144 | 0.257 | I | *Hoplostethus mediterraneus* | 0.572 | 0.137 | 0.291 | I |
| *Dasyatis pastinaca* | 0.011 | 0.989 | 0.000 | I | *Huso huso* | 0.000 | 0.000 | 1.000 | I |
| *Dentex dentex* | 0.090 | 0.134 | 0.775 | I | *Illex coindetii* | 0.609 | 0.210 | 0.181 | I |

Table S1: .../continues from the previous page.

| **species** | **A1 (O)** | **A2 (E)** | **A3 (P)** | **Or** | **species** | **A1 (O)** | **A2 (E)** | **A3 (P)** | **Or** |
| --- | --- | --- | --- | --- | --- | --- | --- | --- | --- |
| *Katsuwonus pelamis* | 0.199 | 0.108 | 0.693 | I | *Pagellus acarne* | 0.505 | 0.000 | 0.495 | I |
| *Knipowitschia caucasica* | 0.895 | 0.105 | 0.000 | I | *Pagellus bogaraveo* | 0.490 | 0.010 | 0.499 | I |
| *Labrus merula* | 0.358 | 0.000 | 0.642 | I | *Pagellus erythrinus* | 0.365 | 0.000 | 0.635 | I |
| *Labrus mixtus* | 0.353 | 0.283 | 0.364 | I | *Pagrus major* | 0.256 | 0.166 | 0.578 | I |
| *Labrus viridis* | 0.300 | 0.335 | 0.365 | I | *Pagrus pagrus* | 0.345 | 0.039 | 0.615 | I |
| *Lagocephalus sceleratus* | 0.432 | 0.053 | 0.516 | L | *Parablennius sanguinolentus* | 0.842 | 0.000 | 0.157 | I |
| *Lagocephalus spadiceus* | 0.549 | 0.032 | 0.420 | L | *Parapenaeus longirostris* | 0.695 | 0.038 | 0.267 | I |
| *Lagocephalus suezensis* | 0.667 | 0.039 | 0.294 | L | *Parexocoetus mento* | 0.872 | 0.128 | 0.000 | L |
| *Lepidopus caudatus* | 0.183 | 0.246 | 0.571 | I | *Phycis blennoides* | 0.281 | 0.000 | 0.719 | I |
| *Lepidorhombus boscii* | 0.457 | 0.000 | 0.543 | I | *Phycis phycis* | 0.366 | 0.144 | 0.490 | I |
| *Lepidotrigla cavillone* | 0.657 | 0.289 | 0.055 | I | *Platichthys flesus* | 0.444 | 0.000 | 0.556 | I |
| *Lepidotrigla dieuzeidei* | 0.633 | 0.326 | 0.041 | I | *Polyprion americanus* | 0.032 | 0.000 | 0.968 | I |
| *Lichia amia* | 0.179 | 0.241 | 0.580 | I | *Pomatomus saltatrix* | 0.213 | 0.239 | 0.548 | I |
| *Lithognathus mormyrus* | 0.457 | 0.000 | 0.543 | I | *Pomatoschistus marmoratus* | 0.964 | 0.016 | 0.020 | I |
| *Liza aurata* | 0.568 | 0.201 | 0.231 | I | *Psetta maxima* | 0.230 | 0.000 | 0.770 | I |
| *Liza carinata* | 0.861 | 0.063 | 0.076 | L | *Pseudocaranx dentex* | 0.185 | 0.000 | 0.815 | I |
| *Liza haematocheila* | 0.469 | 0.000 | 0.531 | I | *Pteromylaeus bovinus* | 0.108 | 0.660 | 0.232 | I |
| *Liza ramada* | 0.599 | 0.000 | 0.401 | I | *Raja asterias* | 0.349 | 0.566 | 0.085 | I |
| *Liza saliens* | 0.577 | 0.000 | 0.423 | I | *Raja clavata* | 0.161 | 0.839 | 0.000 | I |
| *Loligo forbesii* | 0.487 | 0.328 | 0.185 | I | *Raja miraletus* | 0.268 | 0.732 | 0.000 | I |
| *Loligo vulgaris* | 0.516 | 0.484 | 0.000 | I | *Sarda sarda* | 0.256 | 0.141 | 0.603 | I |
| *Lophius budegassa* | 0.134 | 0.472 | 0.394 | I | *Sardina pilchardus* | 0.555 | 0.000 | 0.445 | I |
| *Melikertus kerathurus* | 0.854 | 0.000 | 0.146 | I | *Sardinella aurita* | 0.557 | 0.057 | 0.386 | I |
| *Merlangius merlangus euxinus* | 0.509 | 0.010 | 0.481 | I | *Sargocentron rubrum* | 0.589 | 0.411 | 0.000 | L |
| *Merluccius merluccius* | 0.103 | 0.310 | 0.586 | I | *Sarpa salpa* | 0.604 | 0.000 | 0.395 | I |
| *Micromesistius poutassou* | 0.289 | 0.119 | 0.591 | I | *Saurida undosquamis* | 0.395 | 0.087 | 0.518 | L |
| *Mugil cephalus* | 0.425 | 0.000 | 0.574 | I | *Sciaena umbra* | 0.321 | 0.052 | 0.627 | I |
| *Mullus barbatus* | 0.566 | 0.000 | 0.434 | I | *Scomber japonicus* | 0.347 | 0.000 | 0.653 | I |
| *Mullus surmuletus* | 0.473 | 0.114 | 0.412 | I | *Scomber scombrus* | 0.383 | 0.000 | 0.617 | I |
| *Muraena helena* | 0.039 | 0.342 | 0.619 | I | *Scomberomorus commerson* | 0.001 | 0.540 | 0.459 | L |
| *Mustelus asterias* | 0.151 | 0.849 | 0.000 | I | *Scophthalmus rhombus* | 0.305 | 0.000 | 0.695 | I |
| *Mustelus mustelus* | 0.146 | 0.854 | 0.000 | I | *Scorpaena notata* | 0.551 | 0.040 | 0.409 | I |
| *Myliobatis aquila* | 0.068 | 0.679 | 0.253 | I | *Scorpaena porcus* | 0.353 | 0.281 | 0.366 | I |
| *Nephrops norvegicus* | 0.702 | 0.068 | 0.230 | I | *Scorpaena scrofa* | 0.185 | 0.245 | 0.570 | I |
| *Oblada melanura* | 0.485 | 0.000 | 0.515 | I | *Scyliorhinus canicula* | 0.222 | 0.778 | 0.000 | I |
| *Octopus vulgaris* | 0.525 | 0.021 | 0.453 | I | *Sepia elegans* | 0.788 | 0.212 | 0.000 | I |
| *Oedalechilus labeo* | 0.639 | 0.000 | 0.361 | I | *Sepia officinalis* | 0.555 | 0.434 | 0.011 | I |

Table S1: .../continues from the previous page.

| **species** | **A1 (O)** | **A2 (E)** | **A3 (P)** | **Or** | **species** | **A1 (O)** | **A2 (E)** | **A3 (P)** | **Or** |
| --- | --- | --- | --- | --- | --- | --- | --- | --- | --- |
| *Sepia orbignyana* | 0.607 | 0.393 | 0.000 | I | *Synodus saurus* | 0.295 | 0.304 | 0.400 | I |
| *Seriola dumerili* | 0.185 | 0.132 | 0.683 | I | *Thunnus thynnus* | 0.000 | 0.107 | 0.893 | I |
| *Seriola fasciata* | 0.246 | 0.148 | 0.606 | I | *Torpedo marmorata* | 0.129 | 0.762 | 0.109 | I |
| *Serranus cabrilla* | 0.497 | 0.139 | 0.364 | I | *Torpedo nobiliana* | 0.000 | 0.986 | 0.013 | I |
| *Serranus hepatus* | 0.616 | 0.110 | 0.274 | I | *Torpedo torpedo* | 0.098 | 0.902 | 0.000 | I |
| *Serranus scriba* | 0.423 | 0.043 | 0.534 | I | *Trachinotus ovatus* | 0.503 | 0.388 | 0.109 | I |
| *Siganus luridus* | 0.711 | 0.000 | 0.289 | L | *Trachinus draco* | 0.311 | 0.414 | 0.275 | I |
| *Siganus rivulatus* | 0.762 | 0.000 | 0.238 | L | *Trachinus radiatus* | 0.262 | 0.445 | 0.293 | I |
| *Solea impar* | 0.539 | 0.012 | 0.449 | I | *Trachurus mediterraneus* | 0.435 | 0.009 | 0.556 | I |
| *Solea lascaris* | 0.504 | 0.032 | 0.464 | I | *Trachurus trachurus* | 0.404 | 0.100 | 0.496 | I |
| *Solea solea* | 0.345 | 0.000 | 0.655 | I | *Trigla lucerna* | 0.376 | 0.019 | 0.605 | I |
| *Solenocera membranacea* | 0.774 | 0.065 | 0.161 | I | *Trigloporus lastovisa* | 0.448 | 0.188 | 0.364 | I |
| *Sparisoma cretense* | 0.592 | 0.000 | 0.408 | I | *Trisopterus minutus* | 0.532 | 0.176 | 0.292 | I |
| *Sparus aurata* | 0.217 | 0.000 | 0.783 | I | *Upeneus moluccensis* | 0.613 | 0.028 | 0.358 | L |
| *Sphyraena chrysotaenia* | 0.488 | 0.007 | 0.505 | L | *Upeneus pori* | 0.651 | 0.000 | 0.349 | L |
| *Sphyraena flavicauda* | 0.517 | 0.000 | 0.483 | L | *Uranoscopus scaber* | 0.392 | 0.240 | 0.368 | I |
| *Sphyraena sphyraena* | 0.428 | 0.048 | 0.524 | I | *Xiphias gladius* | 0.000 | 0.395 | 0.605 | I |
| *Spicara flexuosa* | 0.517 | 0.246 | 0.237 | I | *Xyrichthys novacula* | 0.562 | 0.270 | 0.169 | I |
| *Spicara maena* | 0.482 | 0.448 | 0.069 | I | *Zeus faber* | 0.202 | 0.382 | 0.415 | I |
| *Spicara smaris* | 0.663 | 0.000 | 0.337 | I | *Zosterisessor ophiocephalus* | 0.682 | 0.000 | 0.318 | I |
| *Spondyliosoma cantharus* | 0.404 | 0.174 | 0.422 | I |  |  |  |  |  |
| *Squalus acanthias* | 0.000 | 0.864 | 0.136 | I |  |  |  |  |  |
| *Squalus blainville* | 0.175 | 0.768 | 0.057 | I |  |  |  |  |  |
| *Squilla mantis* | 0.656 | 0.195 | 0.149 | I |  |  |  |  |  |
| *Stephanolepis diaspros* | 0.745 | 0.000 | 0.255 | L |  |  |  |  |  |
| *Symphodus cinereus* | 0.686 | 0.000 | 0.314 | I |  |  |  |  |  |
| *Symphodus doderleini* | 0.762 | 0.000 | 0.238 | I |  |  |  |  |  |
| *Symphodus mediterraneus* | 0.659 | 0.036 | 0.305 | I |  |  |  |  |  |
| *Symphodus melanocercus* | 0.773 | 0.000 | 0.227 | I |  |  |  |  |  |
| *Symphodus melops* | 0.576 | 0.070 | 0.355 | I |  |  |  |  |  |
| *Symphodus ocellatus* | 0.746 | 0.000 | 0.254 | I |  |  |  |  |  |
| *Symphodus roissali* | 0.626 | 0.000 | 0.374 | I |  |  |  |  |  |
| *Symphodus rostratus* | 0.785 | 0.011 | 0.203 | I |  |  |  |  |  |
| *Symphodus tinca* | 0.495 | 0.000 | 0.505 | I |  |  |  |  |  |
| *Syngnathus abaster* | 0.683 | 0.263 | 0.054 | I |  |  |  |  |  |
| *Syngnathus acus* | 0.632 | 0.292 | 0.076 | I |  |  |  |  |  |
| *Syngnathus typhle* | 0.406 | 0.570 | 0.023 | I |  |  |  |  |  |

Table S2: The niche overlap between 205 species of Mediterranean fisheries resources examined, specifically for (a) habitat use, (b) food resources of adult/mature stages (c) spawning habitat use. Also, in this table combinations of traits by resource that are not in use from any of the 205 species analysed, are indicated (empty/possibly available niches) Available at:

<https://figshare.com/articles/Koutsidi_Moukas_Tzanatos_Table_S2_xlsx/12197991>
